# Analysis on Effectiveness of Impact Based Heatwave Warning Considering Severity and Likelihood of Health Impacts in Seoul, Korea

**DOI:** 10.3390/ijerph18052380

**Published:** 2021-03-01

**Authors:** Yeora Chae, Jongchul Park

**Affiliations:** Korea Environment Institute, 370 Sicheong-daero, Sejong 30147, Korea; yrchae@kei.re.kr

**Keywords:** heatwave, impact forecast, severity, warning system, threshold temperature

## Abstract

Many countries are operating a heatwave warning system (HWWS) to mitigate the impact of heatwaves on human health. The level of heatwave warning is normally determined by using the threshold temperature of heat-related morbidity or mortality. However, morbidity and mortality threshold temperatures have not been used together to account for the severity of health impacts. In this study, we developed a heatwave warning system with two different warning levels: Level-1 and Level-2, by analyzing the severity and likelihood of heat-related morbidity and mortality using the generalized additive model. The study particularly focuses on the cases in Seoul, South Korea, between 2011 and 2018. The study found that the threshold temperature for heat-related morbidity and mortality are 30 °C and 33 °C, respectively. Approximately 73.1% of heat-related patients visited hospitals when temperature was between 30 °C and 33 °C. We validated the developed HWWS by using both the threshold temperatures of morbidity and mortality. The area under curves (AUCs) of the proposed model were 0.74 and 0.86 at Level-1 and Level-2, respectively. On the other hand, the AUCs of the model using only the mortality threshold were 0.60 and 0.86 at Level-1 and Level-2, respectively. The AUCs of the model using only the morbidity threshold were 0.73 and 0.78 at Level-1 and Level-2, respectively. The results suggest that the updated HWWS can help to reduce the impact of heatwaves, particularly on vulnerable groups, by providing the customized information. This also indicates that the HWWS could effectively mitigate the risk of morbidity and mortality.

## 1. Introduction

South Korea, Japan, China, Australia, and many countries in EU have experienced record-breaking heatwaves, especially in 2018 and 2019 [1,2,3,4,5]. It is expected that the intensity, duration, and frequency of heatwaves will increase globally due to climate change [6]. Additionally, the length of the longest heatwave event is projected to persist for more than a week and possibly up to a month in the late 21st century over the Korean Peninsula [7].

Since the extreme heat event is expected to affect human health [8,9,10,11,12], many countries have developed the heatwave warning systems (HWWSs) to reduce its impact [13,14,15,16,17,18]. A few studies have shown that the HWWSs have contributed to the reduction of excess mortality. In Philadelphia, USA, it was reported that the HWWS could have reduced the deaths of about 117 people from heatwaves during the period 1995–1998 [19]. In France, after the 2003 European heatwave, the Heat Health Watch Warning System was established. In the 2006 heatwave, 2065 deaths were seen, which was smaller than the 6452 deaths predicted based on an epidemiological model for 1975–2003 [20]. The Korea Meteorological Administration (KMA) has also developed its own Heat Watch Warning System, which has been operating since 2008 [9].

The HWWS should be able to predict the impact of heatwaves on human health from mild to severe and provide countermeasures. The severity of the health impact of heatwaves varies from outpatient care to hospitalization and death. Several previous studies have shown how heatwaves are related to mortality, outpatient care, or hospitalization. For example, the increasing temperature raises the number of outpatients with heat-related illness (HRI) [21], emergency room admissions [22], and hospitalization due to mental illness [8]. The measures responding to heatwaves will change depending on the severity of their impact. If the number of outpatients with only mild symptom tends to increase, they could be treated by the efforts of each individual. However, if the number of severe patients or death rises, the government needs to intervene and provide its own measures [23].

Most heatwave advisory and warning systems classify the warning level based on the epidemiological relationship between temperature and all causes of excess mortality [13,14,24,25,26,27]. The external causes are, however, excluded. Meanwhile, in Japan, a preventive information system is established based on heat stroke [28]. The existing HWWS generally uses only one health proxy to determine the threshold temperature for warning. Thus, there is a question whether this current HWWS can effectively warn for the various impacts of heatwaves on human health. Appendix A Table A1 contains the health impact proxies and alert issuance methods used by HWWS in each country.

In England, it has been pointed out that the HWWS has limitations in the prevention of HRI because most of the patients with HRI occur at low warning levels (Level-0 and Level-1) [29]. This suggests the possibility that the warning system, which is based on excess mortality, will not be effective in warning of HRI. Direct evidence is limited, but several studies have shown the possibility of different threshold temperatures for morbidity and mortality. In London and Eastern England, all-cause emergency admissions increased under a daily mean temperature above 13.5 °C, while all-cause mortality increased at about 16 °C [29]. Some studies have shown that excess mortality from heat waves in London increased dramatically at about 19 °C [30,31]. Emergency department visits (2011~2013) in Shanghai, China increased at 25 °C on daily mean temperature [32]. By comparison, mortality (1996~2008) increased at about 27.5 °C [33]. In North Carolina, USA, emergency department visits for heat-related illness (2007~2008) increased sharply at about 26 °C [34], and mortality (2007~2011) increased at 28 °C [35]. Since the response to temperature has temporal variation [29,36], it is possible that the threshold temperature varies depending on the duration of the data used and the statistical method. Therefore, there is a limit to directly comparing the threshold temperatures analyzed in different studies. However, it follows from what has been said that there may be differences in threshold temperature depending on the severity.

The South Korean government also introduced the Heat Watch Warning System based on the threshold temperature. The threshold temperature is determined by using the results of an epidemiological study [37,38,39]. Park et al. [38] analyzed the relationship between excess mortality and temperature in Seoul and proposed the possible threshold temperatures. The KMA constructed two different warning levels, Level-1 and Level-2, for heatwaves based on threshold temperatures. The threshold temperatures are set by the daily maximum temperature. If the daily maximum temperature exceeds 33 °C, it is Level-1. If the maximum temperature exceeds 35 °C, the warning level turns to Level-2 [40]. However, based on the best research, we found none of the studies have looked into whether this system is effective to prevent heat-related morbidity or mortality.

The main goal of this study is to analyze whether the HWWS using the threshold temperature can properly warn for heat-related morbidity and mortality. To do that, we set two different types of threshold temperatures based on all-cause mortality and heat-related morbidity. Then, we analyzed how the threshold temperature is associated with heat-related morbidity and excess mortality.

## 2. Materials and Methodology

### 2.1. Research Scope

The study area was Seoul, South Korea where the threshold temperature used in the Heat Watch Warning System was developed. The morbidity, mortality, and weather data for the period 2011–2018 summer (June to August) were used. During the same period, the daily maximum temperature in Seoul was 29.1 °C on average. The hottest year was 2018, and the average daily maximum temperature was 30.5 °C.

**Figure 1:**
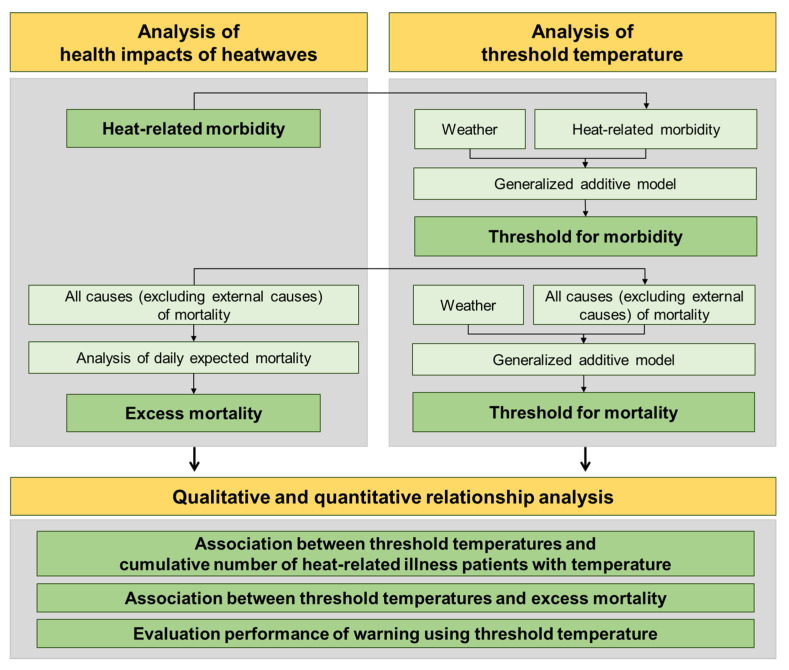
Flowchart of the study procedure.

The study procedure is shown in Figure 1. First, heat-related morbidity and excess mortality were analyzed as proxies of health impacts from heatwaves. Next, a threshold temperature for heat-related morbidity and a threshold temperature for mortality were analyzed. Finally, the association between threshold temperatures and health impact proxies were analyzed in three ways. (1) The cumulative number of HRI patients with increasing temperature was compared to the two threshold temperatures. (2) The daily excess mortality was also qualitatively compared to the two threshold temperatures. (3) The performance of warning based on threshold temperature was evaluated using the model skill score.

### 2.2. Materials

Population of heat-related morbidity was obtained from Healthcare Bigdata of National Health Insurance Sharing Service of Korea. Mortality data were obtained from Statistics Korea’s data on causes of deaths. All causes (excluding external causes) of mortality were used in this study; the ICD-10 (International Statistical Classification of Diseases-10th Revision) codes are from A to R. These data cover the period of 2011–2018.

HRI monitoring data obtained from the Korea Disease Control and Prevention were used as verification data to evaluate the performance of threshold temperature. Since Healthcare Bigdata includes all patients who visited the hospital, patients who were not affected by the heatwave are included as noise. On the other hand, the monitoring data are patients who were determined to have been affected by heatwaves among emergency room visitors by emergency medicine doctors. Therefore, we used this data as verification data in the performance analysis. All-cause mortality or death from certain diseases (e.g., cardiovascular, respiratory, and cancer) have been adopted in heatwave studies [41,42,43,44,45,46]. This study adopted all-cause mortality in the study.

Weather data was obtained by downscaling to a 1 km spatial resolution the daily maximum temperature (Tmax), daily minimum temperature (Tmin), daily average relative humidity, and daily mean windspeed from the digital forecasts provided by the Korea Meteorological Administration (KMA). The Gaussian process regression model was used for downscaling [47]. Durations of heatwaves, tropical nights, and dry days were also used in this study. The heatwave was defined as the 90th percentile of Tmax during the period ranging from 2011 to 2018. The tropical night had a Tmin of 25 °C or higher. Days with precipitation below 1 mm/day were regarded as dry days.

### 2.3. Statistical Methods

A generalized additive model (GAM) was employed as a statistical method to quantitatively analyze the relationship between temperature and health. GAMs have been widely used in time-series research because they can express the influence of nonlinear confounding variables in a nonlinear manner using nonparametric functions [36,48]. The statistic model used in this study was expressed as follows:

(1) $$\ln\left(E\left(Y\right)\right)=\beta_{0}+s\left(T_{\max}\right)+s\left(H_{\mathrm{avg}}\right)+\mathrm{wind}+\mathrm{hw}+\mathrm{tn}+\;\mathrm{dry}+s\left(\mathrm{DOY}\right)+s\left(\mathrm{sn},\;k=2*8\right)+f\left(W\right)+f\left(\mathrm{yr}\right),$$

where E(Y) denotes the expected daily death counts in the mortality cases and patient counts in the morbidity cases; s(T_max_) is the daily maximum temperature; s(H_avg_) is the daily average humidity; wind is the daily average wind speed; hw is duration of heatwaves; tn is duration of tropical nights; dry is the duration of dry days; s(DOY) is the day of year; and s(sn) is the serial number of the date. The s denotes smooth, nonparametric functions. f(W) and f(yr) represent the day of the week and year, respectively, and were used as dummy variables. The day of the week was classified to three types (holidays, the day after the holiday, and other days). R 3.4.0 and the mgcv package were used for the GAM analysis. The threshold temperature was determined from smoothed curves of the GAM.

Daily excess mortality was estimated as the difference between daily deaths and expected deaths. The expected daily deaths were calculated using the following Equation (2). This formula is based on the formula proposed by Jeong et al. [49] and applies the method of FluMOMO v4.2 [50], which considers the population structure.

(2) $$E\left(y,d\right)=\overset{2}{\underset{i=1}{\sum }}\overset{\mathrm{ages}}{\underset{i}{\sum }}E\left(y,d\right)\mathrm{ij}$$

E(y,d)ij = Mij × W(d)ij × Wij(y, w) × Wij(y)(3)

where E(y,d) is the number of expected deaths on the d-th day of year y, and E(y,d)ij is the number of expected deaths by gender (i) and age (j) on a given date. Age groups were divided at 5-year intervals, and those over 65 were categorized as one group. Mij is the average number of deaths per day for each gender and age group over the entire period. Wij(d) is the value obtained by dividing the number of deaths on the d-th day by Mij. When calculating Wij(d), a 7-day weighted moving average was applied to remove weekly variability. Weights 1, 2, 3, 4, 3, 2, and 1 were applied in 7 days. The highest weight is assigned to the days to be calculated. Wij(y, w) is the number of deaths on the w (day of the week) divided by the average number of deaths on the w in the y year. Wij(y) is the total number of deaths in year y divided by the annual average number of deaths in the entire period.

### 2.4. Evaluation Methods Using Skill Score

The area under the curve (AUC) was used as the skill score for evaluation. The AUC was calculated from the receiver operating characteristic (ROC) curve, drawn using the sensitivity (true positives/(true positives + false negatives)) and the specificity ((true negatives/(false positives + true negatives)) derived from the confusion matrix [51]. The R package pROC was used to calculate AUC [52].

The predicted event in the confusion matrix was the individual warning level, and the actual event was defined as a gradual increase in the number of patients or the occurrence of mortality. The warning to be evaluated consisted of two levels (Level-1 and Level-2). Level 1 is a period in which warning regarding an increase in the HRI morbidity is required, and level-2 is a period in which a warning regarding an increase in mortality is required.

## 3. Results

### 3.1. Threshold Temperatures and HRI Patients

**Figure 2:**
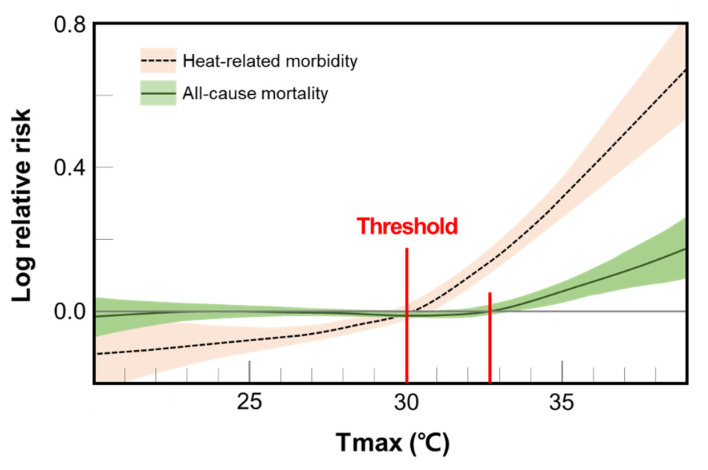
Association between daily maximum temperature (Tmax) and negative health outcomes (all-cause mortality and heat-related morbidity).

The effects of maximum temperature on heat-related morbidity and mortality showed a pattern as shown in Figure 2. The heat-related morbidity had a pattern of increasing rapidly from a relatively lower temperature than mortality. The threshold temperature for heat-related morbidity was 30 °C, while the threshold for mortality was approximately 33 °C.

**Figure 3:**
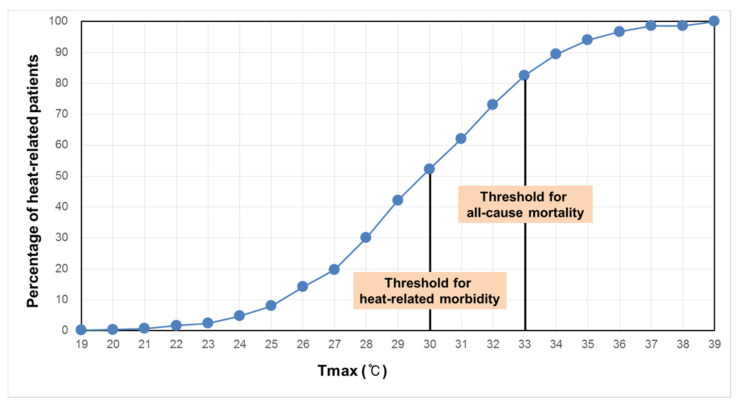
Cumulative number of heat-related morbidities with temperature increase.

**Figure 4:**
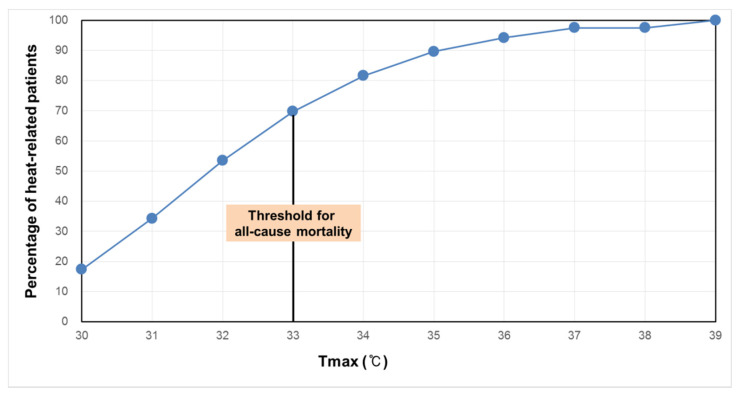
Cumulative number of heat-related morbidities with temperature increase above 30 °C, which is the threshold temperature for heat-related morbidity.

The comparison between the cumulative number of heat-related morbidity with respect to the two threshold temperatures shows that most of the heat-related morbidity already occurred before the temperature reached 33 °C (Figure 3), which is the threshold temperature for mortality. Even if the cumulative number of heat-related morbidities was only counted above 30 °C, 53.5% of heat-related morbidity occurred before 33 °C (Figure 4).

England, France, and the US issue warnings when the threshold temperature will be exceeded within the next few days [14,15,24,26,27]. This method can also be considered in Korea. Currently, Korea issues a warning when the mortality threshold temperature has been exceeded for two consecutive days. Therefore, there is a need for a way to issue a warning about an increase in heat-related illness before reaching the mortality threshold temperature. If a lower warning level is issued a few days before reaching the threshold temperature, is it possible to cover the heat-related morbidity that occurs before the threshold temperature of mortality is reached?

Table 1 shows that this is difficult. The number of days when the daily maximum temperature exceeded 30 °C and was below 33 °C was 194 during 2011 to 2018, in Seoul. If a warning was issued 2 days before the temperature reached 33 °C, the total number of days for which the warning was issued was 54. However, these days include 22 instances when the temperature did not exceed 30 °C. Therefore, the number of days for which warnings were issued when the temperatures ranged between 30 °C and 33 °C was only 32. Approximately 84% of the days when the daily maximum temperature was between the morbidity threshold and the mortality threshold were missed. As a result, it is highly likely that warning caused by the threshold temperature of mortality is not effective in preventing heat-related morbidity.

### 3.2. Threshold Temperatures and Excess Mortality

Excess mortality tended to increase on average above the mortality threshold temperature. The average of excess mortality was 5.79 per day above 33 °C. On the other hand, the average of excess mortality was −0.02 between the daily maximum temperatures from 30 °C to 33 °C (Table 2). The difference of the two average values was statistically significant (*p*-value = 0.0013). The 75th percentile of excess mortality above the mortality threshold temperature was about three times higher than the value above the morbidity threshold temperature. The maximum excess mortality (44.79) above the mortality threshold temperature was also approximately twice the value of the excess mortality (24.54) that occurred after the morbidity threshold temperature.

This result suggests that warnings based on the threshold temperature of heat-related morbidity overestimates the risk of mortality by heatwaves. Frequent issuance of warnings may reduce the effectiveness of the warning by increasing the fatigue of the consumer receiving the information.

**Figure 5:**
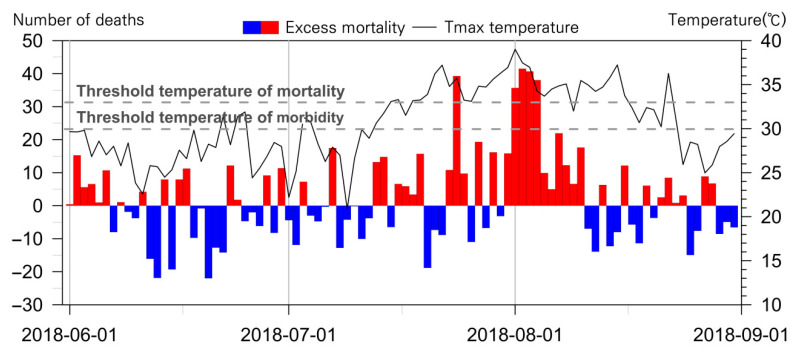
Comparison of daily excess mortality and the two threshold temperatures in Seoul, 2018.

The graph comparing the daily excess mortality occurrence and the two threshold temperatures shows that the threshold temperature for heat-related morbidity has a limit to the warning of the risk of mortality. In Figure 5, the bar graph indicates the excess mortality. The red bars indicate the days when the number of deaths was greater than the expected deaths. Blue bars indicate fewer deaths than expected. The days when excess mortality increased dramatically appeared in 24 July, and between 1 and 4 August. During this period, the daily maximum temperature remained above 33 °C. It was difficult to find a definite increase in excess mortality within the temperatures between 30 °C and 33 °C. The threshold temperature of heat-related morbidity is difficult to use to warn of the risk of death from heatwaves.

### 3.3. Performance Comparison of Heatwave Early Warnings

According to the comparison of actual and predicted HRI occurrences, the AUC was improved when the two threshold temperatures were combined compared to using the threshold temperature for either mortality or morbidity. Table 3 shows the evaluation results of warnings for Level-1 and Level-2 according to the type of threshold temperature. The actual events were HRI patients and deaths monitored in emergency departments. Level-1 was defined as the day on which the number of patients with HRI increased (50th to 90th percentile patients) to evaluate the performance of threshold temperature against morbidity. Level-2 was defined as the day on which deaths from HRI occurred or the number of patients with HRI increased to more than 90th percentile to evaluate the performance of the mortality threshold temperature.

The AUCs for the mortality threshold temperature were 0.60 and 0.86 at Level-1 and Level-2, respectively. When the morbidity threshold temperature was used, the AUC for Level-1 increased to 0.73, but the AUC decreased to 0.78 for Level-2. When the two threshold temperatures were combined, the AUCs were 0.74 and 0.86, respectively (Table 3). The combination of the two threshold temperatures showed the best performance in the early warning of heatwaves.

**Figure 6:**
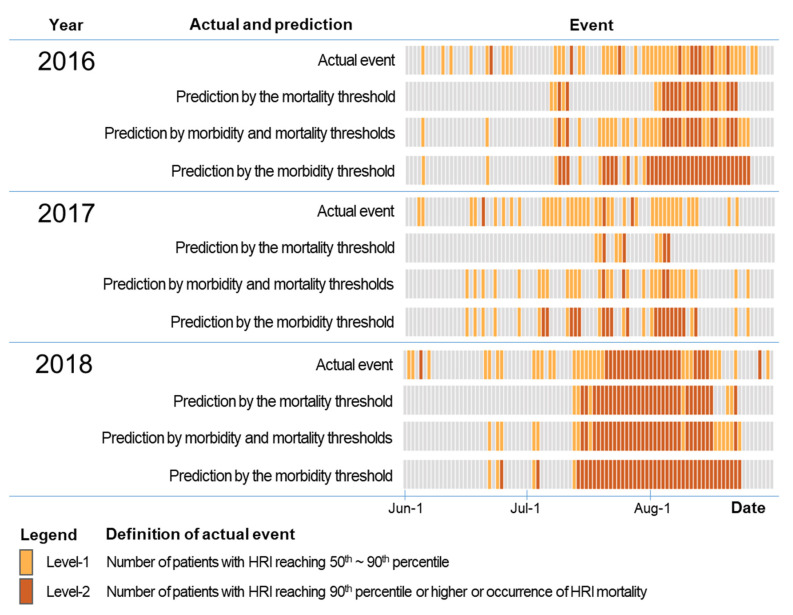
Comparison of actual and predicted heat-related illness (HRI) events: the actual event was based on monitoring data in the emergency department of the hospital; predicted events were determined according to types of the threshold.

The daily comparison of the actual and predicted events showed that the frequency of false negative (actual: yes, predicted: no) for Level-1 was high when the mortality threshold temperature was used. The prediction failure for Level-1 can be clearly seen in the 2016 and 2017 cases. In the case of using only the morbidity threshold temperature, there were many false positives (actual: no, predicted: yes) for Level-2. There was a pronounced tendency in 2016 and 2018 to overestimate Level-2. When both threshold temperatures were used, reasonable results were obtained in the prediction of Level-1 and Level-2 compared to when using a single threshold temperature (Figure 6).

## 4. Conclusions

This study showed the possibility that the warning system based on excess mortality may have limitations in reducing heat-related morbidity. The KMA is issuing warnings for heatwaves of Level-1 and Level-2, respectively, using threshold temperatures of 33 °C and 35 °C. According to our findings, these criteria are ineffective in the prevention of heat-related morbidity because the threshold temperatures are designed based on mortality. Most cases of patients with heat-related illness have already occurred before reaching Level-1. Furthermore, this study showed that the warning system based on heat-related morbidity would have limitations when warning about excessive mortality. The reason can be found in the difference in threshold temperature for morbidity and mortality.

This study suggests that, for the heatwave warning system to contribute to reducing both patient admissions and casualties, it is necessary to combine the threshold temperature of morbidity and mortality. The combination of morbidity and mortality thresholds showed improved performance compared to a single threshold temperature. Therefore, it is possible to improve the effectiveness of the warning system by identifying the lower (higher) threshold based epidemiological studies of morbidity (mortality).

This study has apparent limitations in that we only dealt with the case of Seoul. Evidence may need to be supplemented with more case studies in the future. Nevertheless, this study is significant because it raised and demonstrated issues of the heatwave warning system developed using a single health impact proxy. The results of this study can contribute to improving the current Heat Watch Warning System of Korea by providing warnings for morbidity as well as mortality.

## Figures and Tables

**Table 1:** Number of days on which warning was issued in Seoul, from 2011 to 2018 (based on the assumption that a warning was issued from two days before the daily maximum temperature reached the threshold temperature of mortality (33 °C)).

Type of Day	Number of Days
① Number of days when the daily maximum temperature ≥30 °C and <33 °C	194
② Number of days when the warning was issued	54
③ Number of days when the daily maximum temperature <30 °C in cases ②	22
④ Number of days missed from the warning in cases ① (① − (② − ③))	162

**Table 2:** Number of excess deaths per day by threshold temperature in 2011–2018.

Quartile	30 ≤ T_max_ < 33 (*n* = 195)	33 ≤ T_max_ (*n* = 81)
Max	24.54	44.79
75%	5.89	14.06
Mean	−0.02	5.79
25%	−5.88	−6.47
Min	−26.43	−21.56

**Table 3:** Evaluation result using AUC of heatwave early warning by threshold types.

Threshold Type	AUC		Warning Criteria
	Level-1	Level-2	
Mortality	0.60	0.86	Level-1: The mortality threshold will be exceeded within the next 2 days Level-2: The mortality threshold has been exceeded
Morbidity	0.73	0.78	Level-1: The morbidity threshold has been exceeded Level-2: The morbidity threshold has been exceeded for at least two days
Morbidity and mortality	0.74	0.86	Level-1: The morbidity threshold has been exceeded Level-2: The mortality threshold has been exceeded

Level-1: number of patients with HRI reaching 50th~90th percentile. Level-2: number of patients with HRI reaching 90th percentile or higher or occurrence of HRI mortality.

## Appendix A

**Table A1:** Classification of levels and health impact proxies in heatwave warning systems (HWWS).

Country	HWWS	Warning Level	Criteria for Warnings	Health Impact Proxy	Reference
Australia	Heat health alert system	1	Alert: When forecast daily average temperatures are predicted to reach or exceed the heat health temperature threshold.	Heat-related illness	[53,54,55]
England	Heat-health alert service	1~4	Level 2: When there is a 60% risk of heatwave in the next 2 to 3 days. Level 3: When threshold temperatures have been reached in any one region or more.	Excess mortality	[24]
France	Heat Health Watch Warning System	1~4	Level 2: When thresholds are to be reached within three days. Level 3: When the thresholds are reached.	Excess mortality	[14,26,27,56]
Japan	Heatstroke prevention information	1~4	Levels 1 to 4 will be issued, when WBGT is more than 21, 25, 28, and 31 °C, respectively.	Heat stroke	[28]
South Korea	Heat Watch Warning System	1 & 2	Level 1: When maximum temperature is more than 33 °C for at least two consecutive days. Level 2: When maximum temperature is more than 35 °C for at least two consecutive days.	Excess mortality	[37,38,39]
USA	Heat Safety	1~4	Excessive Heat Outlooks: When the potential for an excessive heat event in the next 3–7 days is identified. Excessive Heat Watches: When conditions are favorable for an excessive heat event in the next 24 to 72 h. Heat Advisory: Within 12 h of the onset of extremely dangerous heat conditions. The general rule of thumb for this Advisory is when the maximum heat index temperature is expected to be 100°F or higher for at least 2 days, and night time air temperatures will not drop below 75°F; however, these criteria vary across the country, especially for areas that are not considered to exhibit dangerous heat conditions. Excessive Heat Warning: Within 12 h of the onset of extremely dangerous heat conditions. The general rule of thumb for this Warning is when the maximum heat index temperature is expected to be 105°F or higher for at least 2 days and night time air temperatures will not drop below 75°F; however, these criteria vary across the country, especially for areas not used to extreme heat conditions.	Human experimentation	[15]
